# Evaluation of the Accuracy of Estimated Endpoint Titer of NOVA View in Indirect Immunofluorescent Antinuclear Antibody Testing

**DOI:** 10.3390/diagnostics14151580

**Published:** 2024-07-23

**Authors:** Hae Weon Cho, Soon-Ho Jeong, Jun Sung Hong, Dokyun Kim, Yongjung Park, Seok Hoon Jeong

**Affiliations:** 1 Department of Laboratory Medicine, Yonsei University College of Medicine, Seoul 03722, Republic of Korea; kaiencho67@naver.com (H.W.C.); ypark119@yuhs.ac (Y.P.); kscpjsh@yuhs.ac (S.H.J.); 2 Research Institute of Bacterial Resistance, Yonsei University College of Medicine, Seoul 03722, Republic of Korea; 3 Department of Laboratory Medicine, Myongji Hospital, Goyang 10475, Republic of Korea; 4 Department of Laboratory Medicine, Gangnam Severance Hospital, Yonsei University College of Medicine, Seoul 03722, Republic of Korea; soonhoj823@yuhs.ac; 5 Department of Companion Animal Health and Science, Silla University, Busan 46958, Republic of Korea; jumphong2@nate.com; 6 Department of Laboratory Medicine, Yongin Severance Hospital, Yonsei University College of Medicine, Yongin 16995, Republic of Korea

**Keywords:** antinuclear antibody, indirect immunofluorescence assay, automation, serial dilution endpoint titration, single-well titration, antibodies, antinuclear, fluorescent antibody technique, indirect, automation, laboratory

## Abstract

For antinuclear antibody (ANA) screening, the gold standard method is an indirect immunofluorescence assay (IIFA) using HEp-2 cells, and a serial dilution test is needed to determine the endpoint titer. We aimed to evaluate the accuracy of the estimated endpoint titer (eEPT) by the NOVA View system, by comparing it with the EPT by the serial dilution method (dEPT). The endpoint titers of a total of 1518 ANA positive cases with five major patterns including speckled, homogeneous, centromere, nucleolar, and nuclear dots patterns were determined using both the estimation function and the serial dilution method by the NOVA View system. A significant correlation between the light intensity unit (LIU) values and dEPTs was identified in all five patterns with high *ρ* values, ranging from 0.666 to 0.832. However, the overall exact match rate between dEPT and eEPT was 22.1% (336/1518), with the ±one-titer match rate being highest in the centromere pattern (62.8%, 81/129), and lowest in the homogeneous pattern (37.6%, 200/532). This suggests that while LIU values correlate well with dEPT, there are discrepancies in numerical agreement. Most cases that did not show an exact match, showed one-to-three-titer overestimations by eEPT. Therefore, adjusting eEPT downward significantly improved the concordance rates with dEPTs. Further investigation for an appropriate cutoff of LIU values for determining eEPT should be performed for clinical application and contribution to the standardization of the ANA titer.

## 1. Introduction

The detection of antinuclear antibodies (ANAs) is useful for diagnosing various autoimmune diseases such as systemic lupus erythematosus, Sjögren’s syndrome, rheumatoid arthritis, systemic sclerosis, and undifferentiated connective tissue disease [1,2,3], and the gold standard for ANA screening test is an indirect immunofluorescence assay (IIFA) using HEp-2 cells [4,5,6]. The advantages of the ANA IIFA using HEp-2 cells over other methods include high sensitivity, capability of detecting a broad range of autoantibodies, and ability to define spatial distribution patterns and to evaluate the endpoint titer [2,3,4,5], and the immunofluorescence patterns that correspond to the distribution of autoantigens on the cellular components are reported to be useful in specifying the types of autoimmune disease [1,2,3]. Although the correlation between ANA titer and disease activity is unclear, ANA titer could also be a useful parameter to diagnose autoimmune disease due to the rarity of high ANA titers in healthy individuals, and positivity in more than 1:160 dilution increases the specificity of the ANA test for the diagnosis of autoimmune disease [2,3,7]. However, the determination of ANA titers by manual fluorescence microscopic observation is laborious and time-consuming, and the requirement for qualified experts and the subjectivity of interpretation complicate the standardization of ANA IIFA [8,9].

During the last few decades, several commercial automated ANA IIFA systems that can prepare slides, obtain digital images, determine the ANA patterns, and quantify the fluorescence intensities have been introduced: AKLIDES (Medipan, Dahlewitz, Germany), EUROPattern Suite (Euroimmun AG, Luebeck, Germany), HELIOS (Aesku Diagnostics, Wendelsheim, Germany), Image Navigator (Immuno Concepts, Sacramento, CA, USA), NOVA View (Inova Diagnostics, San Diego, CA, USA), and Zenit G-Sight (Menarini Diagnostics, Florence, Italy) [6,10,11]. Automated ANA IIFA systems have advantages in reducing the labor force and working hours and allowing the raw data of the tests to be stored and shared in the form of digital images [11,12,13,14]. Furthermore, these automated ANA IIFA systems are expected to reduce the subjectivity that can arise when medical technologists and pathologists process and interpret clinical specimens [6,10,15].

Among the automated ANA IIFA systems, NOVA View can provide an estimated endpoint titer without serial dilution tests by calculating the intensity of the fluorescence in a 1:80 dilution test, namely, the estimated endpoint titer (eEPT). The eEPT function could reduce the labor and costs for determining the endpoint titer of ANAs and could have the potential to suggest standardized qualitative results; however, few studies have investigated the performance and usefulness of the eEPT of NOVA View [16,17]. In this study, we aimed to evaluate the accuracy of the eEPT estimated by NOVA View in five ANA patterns (speckled, homogeneous, centromere, nucleolar, and nuclear dots) compared to EPT by serial dilution (dEPT) as a reference method.

## 2. Materials and Methods

### 2.1. Study Design

**Figure 1:**
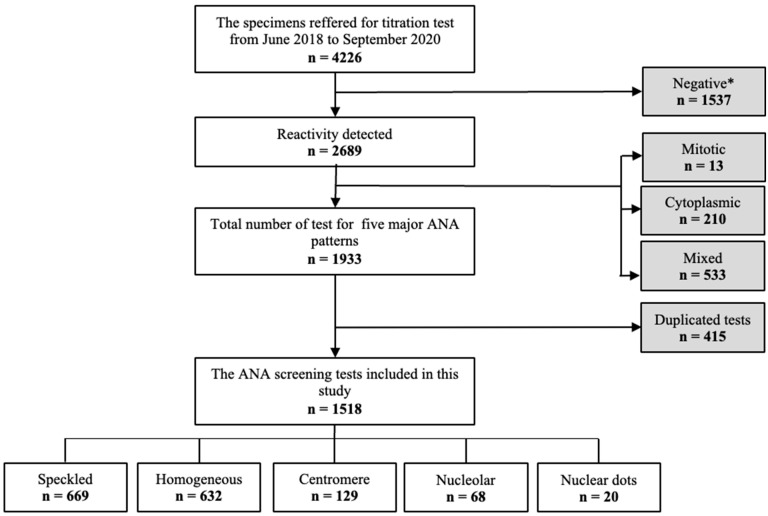
Study design and schematic flow chart of patient inclusion and exclusion. * No reactivity at the dilution of 1:80.

From June 2018 to September 2020, a total of 4226 consecutive serum specimens were referred for routine HEp-2 IIFA titration tests at a teaching hospital in South Korea. Among them, 1537 specimens with no reactivity at the dilution of 1:80 were excluded [3], and 2689 specimens with positive reactivity were evaluated for ANA pattern. Among them, 756 specimens with mitotic (*n* = 13), cytoplasmic (*n* = 210), and mixed (*n* = 533) patterns could not be estimated by NOVA View, and 415 duplicated tests for one patient were excluded from the study. Finally, a total of 1518 cases were included in this study (Figure 1). Patient information, including demographic conditions, was retrieved from electronic medical records. Clinical diagnosis of the patients was collected in the period of one year before to two years after the ANA IIFA tests. All processes of this study were in accordance with the ethical standards of the institutional and national research committee and with the 1964 Helsinki Declaration and comparable ethical standards. The study protocols were approved by the Institutional Review Board of Gangnam Severance Hospital and the need for informed consent for reviewing medical records of the study population was waived due to the purely retrospective nature of this study (approval number: 3-2022-0226).

### 2.2. Detection of ANA and Determination of Endpoint Titer by Serial Dilution and Estimation Function

Specimens were diluted and processed into slides using a QUANTA-Lyser 240 (Inova Diagnostics, Inc., San Diego, CA, USA). ANA was detected by NOVA Lite^®^ HEp-2 ANA with DAPI kit (Inova Diagnostics) using goat anti-human IgG specific fluorescein-5-isothiocyanate (FITC)-labeled conjugate containing 4′-6-diamidino-2-phenylindol (DAPI). ANA images were captured by an Olympus I × 81 inverted IFA microscope with 40× objectives and dual-band DAPI/FITC filters and a Basler camera on the NOVA View instrument system and read by the NOVA View^®^ (Inova Diagnostics) digital IIF microscope (software version 2.0.5.1). DAPI fluorescence was used by NOVA View software for localizing and focusing on HEp-2 cells. Three to five images based on the FITC and DAPI signals for each well on the slide were obtained. Using FITC images, the system can propose the ANA patterns based on software algorithms and measure the average light intensity units (LIUs). The pattern was determined after being confirmed by two experienced clinicians. To determine the dEPT, the specimens were diluted up to 1:640, and two experienced clinicians interpreted titers based on the ANA images obtained by NOVA View. In addition, the eEPTs were determined based on the measured LIU at a dilution of 1:80, up to 1:2560 for speckled and homogeneous patterns, and up to 1:5120 for centromere, nucleolar, and nuclear dots patterns.

### 2.3. Statistical Analysis

Box-and-whisker plots were used to evaluate the relationships of eEPT and dEPT with LIU, and Spearman’s rank correlation coefficient (*ρ*) was used for correlation analyses between dEPT and LIU. The results of eEPT and dEPT were compared by three types of concordance: exact match, ±one-titer match, and categorical match based on the cut-off titer of 1:160, which is the common clinical cut-off for the diagnosis of autoimmune disease (AD). All the statistical analyses were performed using R software version 4.3.1 (R Development Core Team 2023; http://www.R-project.org/). Packages ‘ggpubr’ and ‘ggplot2’ were used for visualization of the statistical analysis results. Statistical significance was established at a *p* value less than 0.05.

## 3. Results

### 3.1. Patient Characteristics

The demographic and clinical characteristics of patients enrolled in this study are summarized in Table 1. Among the 1518 patients, AD was diagnosed in 45.9% (*n* = 697) of patients, and the most common AD was systemic lupus erythematosus (130/1518, 8.6%), followed by Sjögren’s syndrome (*n* = 121, 8.0%), autoimmune hepatitis (*n* = 108, 7.1%), rheumatoid arthritis (*n* = 81, 5.5%), systemic sclerosis (*n* = 35, 2.3%), and primary biliary cholangitis (0.4%, *n* = 6). The median age of the patients with ADs was 53.0 [interquartile range (IQR), 39.0–64.0], which was statistically younger than those without AD (median, 59.0; IQR, 43.0–70.0; *p* value < 0.001). The homogeneous pattern was more commonly identified in patients with non-AD (367/821, 44.7%) than in those with AD (265/697, 38.0%). In contrast, a centromere pattern was more common in patients with AD (73/697, 10.5%) than those with non-AD (56/821, 6.8%). More than half (449/697, 64.4%) of the patients with AD showed high dEPT of autoantibody over 1:160, while 65.5% (538/821) of non-AD patients showed low endpoint titer (1:80).

### 3.2. Relationship between Light Intensity Units and Endpoint Titers

**Figure 2:**
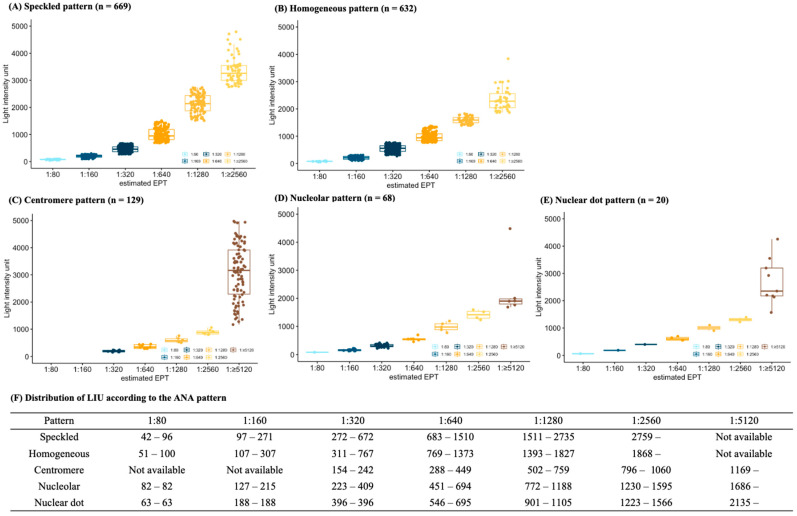
Distribution of light intensity units (LIUs) according to estimated endpoint titers (EPT) in each ANA pattern.

The distribution of LIU according to the eEPT for each ANA pattern is plotted in Figure 2. The exact cut-off values for each eEPT are not disclosed by the manufacturers, but the cut-off values in each pattern were different according to the results of our study. The cut-off values for each dilution in the speckled pattern were as follows: 97 for 1:160, 272 for 1:320, 673 to 683 for 1:640, 1511 for 1:1280, and 2736 to 2759 for 1:2560 dilution. The cut-off values in the homogeneous pattern were lower in 1:80, 1:160, 1:320, and 1:640, and higher in 1:1280 and 1:2560 dilutions than those in the speckled pattern. The cut-off values for the other three patterns could not be specified according to the lack of number of cases.

**Figure 3:**
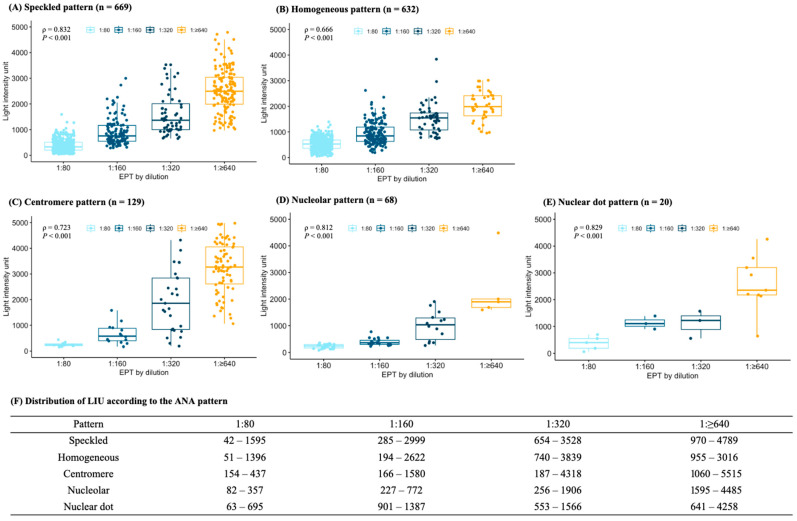
Distribution of LIU according to endpoint titer by serial dilution (dEPT) groups in each ANA pattern.

The distribution of LIUs according to the dEPT showed a large overlap in all patterns (Figure 3). In the speckled pattern, the LIUs were identified between 42 to 1595 in 1:80 dilution, 285 to 2999 in 1:160, 654 to 3528 in 1:320, and 970 to 4789 in 1:640 dilution, and the *ρ* value between LIU and dEPT was 0.832 (*p* < 0.001). The LIU distributions in the homogeneous pattern also showed large overlap among the dEPTs, 51 to 1396 in 1:80, 194 to 2622 in 1:160, 740 to 3839 in 1:320, and 955 to 3016 in 1:640 dilutions, and the *ρ* value was 0.666, which was lower than that in the speckled pattern. Correlation analyses between LIU and dEPT for other ANA patterns also yielded a high *ρ* value of 0.723 for centromere, 0.812 for nucleolar, and 0.829 for nuclear dots patterns with statistical significance.

### 3.3. Concordance of dEPT and eEPT

**Figure 4:**
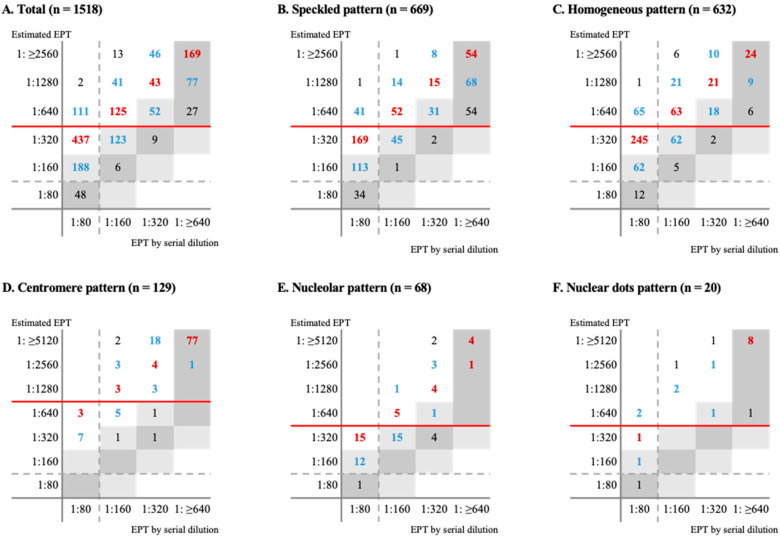
Error grid analysis of concordance between endpoint titer by serial dilution and estimated endpoint titer (eEPT) by NOVA View automated ANA IIFA system: (**A**) Total; (**B**) Speckled pattern; (**C**) Homogeneous pattern; (**D**) Centromere pattern; (**E**) Nucleolar pattern; (**F**) Nuclear dots pattern. The dark grey zones indicate the cases with the same titer between dEPT and eEPT, and the light grey zones indicate the specimens with the ±one-titer difference between dEPT and eEPT. The red-colored numbers indicate the number of cases with the same titer between dEPT by adjusted eEPT, and the blue-colored numbers indicate the number of specimens with the ±one-titer difference between dEPT and adjusted eEPT. The dashed lines indicate the cut-off line based on 1:160 as a cut-off titer and the red colored lines indicate the adjusted cut-off line.

The concordance rate between the eEPTs and dEPTs was calculated in each ANA pattern. As summarized in Table 2, the overall exact match was identified in 22.1%, the ±one-titer match in 46.1%, and the categorical match in 51.4%. The rates of exact match and the ±one-titer match were the highest in the centromere pattern followed by the speckled pattern and nuclear dot pattern, and were the lowest in the homogeneous pattern. Concordance and errors are visualized according to the ANA pattern in Figure 4, and eEPT was always higher than dEPT in all cases.

When adjusting the eEPTs by downgrading two titers from the initial eEPT (i.e., 1:320 to 1:80, 1:640 to 1:160, 1:1280 to 1:320, 1:2560 to 1:640, and 1:≥5120 to 1:≥1280), the rates of exact matches, the ±one-titer matches, and categorical matches showed the greatest improvement. The overall rate of the exact match increased to 51.0%, that of ±one-titer match increased to 93.0%, and that of the categorical match increased to 83.5% (Table 2). For the ANA pattern, speckled, homogeneous, nucleolar, and nuclear dots patterns showed the highest improvement in ±one-titer match rate after two-titer downgraded adjustment, while three-titer downgraded adjustment yielded the highest improvement in the centromere pattern.

## 4. Discussion

The ANA quantitative test is helpful for screening patients with autoimmune disease [2,3]; however, inconsistent results among clinical laboratories can be produced mainly due to inter-individual variation in the interpretation process and lack of objective cut-off [8]. NOVA View offers an automated quantitative interpretation suggesting LIU values, and the clinical relevance of LIU has been reported to reduce intra- and inter-laboratory variance [18,19]. However, the correlation between LIU and dEPT has been rarely evaluated so far, even though the eEPT based on LIU could be useful in producing a rapid and objective ANA endpoint titer. In this study, we analyzed a large number of ANA-positive cases with five major patterns, and the correlation of LIU with dEPT results was evaluated.

The LIU value is an average nuclear fluorescence intensity in nominal units measured by the NOVA View system. The LIU is mostly calculated using the light from antigen–antibody reactions, however, some light from non-specific bonds could be included, which results in an inaccurate correlation between the antigenicity and the LIU value. In addition, a non-linear correlation between the LIU value and the concentration and affinity of autoantibodies can be obtained, and the prozone effect and the difficulty in identifying masked antibodies can lead to the reporting of incorrect ANA titers and/or patterns [13,17,18,19]. In this study, the LIU values and dEPT showed a significant association in all five patterns, consistent with the previous studies [15]; Spearman’s rho values were the highest in the speckled pattern (*ρ* = 0.832) and the lowest in the homogenous pattern (*ρ* = 0.666). Therefore, the LIU could be a useful biomarker for the estimation of endpoint titers.

The algorithm of the eEPT function of the NOVA view system is based on the LIU value, and the cutoff of each titer is not disclosed but is determined according to the built-in standard curves for the eight individual ANA patterns. The analytical performance of eEPT compared with dEPT has not been widely investigated so far. According to the report of Van Hoovels, the rates for an exact match between eEPT and dEPT in the NOVA View system were 51.4%, 54.1%, 44.6%, and 36.2% in homogeneous, speckled, centromere, and nucleolar patterns, respectively, and most of the unmatched cases showed an overestimation of one titer by eEPT [17]. In our study, the overall rates for the exact match of eEPT compared with dEPT were only 22.1% (336/1518), and eEPTs in the homogeneous pattern showed the lowest rate (10.9%, 58/532), and the centromere pattern showed the highest (61.2%, 79/129). Consistent with the previous study [17], most of the cases that did not show an exact match showed one- or two-titer overestimations by eEPT, and the two-titer downgrade modification of eEPT showed the biggest improvement in the rates of the ±one-titer match in speckled, homogeneous, nucleolar, and nuclear dot patterns, with the three-titer downgrade having the biggest improvement in the centromere pattern. Automated ANA IIFA systems other than NOVA View, which is also capable of quantifying fluorescence intensities, have been studied for their potential to predict ANA endpoint titers [14,20,21,22,23]. However, there are still limitations, and further development is needed to replace the conventional dEPT without confirmation by an expert. Further investigation should be performed to determine cut-off values of LIU, which improves concordance rates compared with dEPT, which is considered the gold standard method.

One of the limitations is that this study was implemented in a single country and a single institution, and racial diversity and inter-laboratory variation were not considered. However, our institution participates in the external quality assessment program and is certified as a clinical examination laboratory by the Korean Association of External Quality Assessment Service. In addition, a large number of consecutive specimens of patients in a routine laboratory setting were included in this study to minimize bias, and two separate clinicians interpretated to reduce inter-individual variation. In addition, the limited number of patients with nuclear and nuclear dots patterns is another limitation of this study, so further studies are needed to suggest a more accurate adjusted titer.

## 5. Conclusions

In conclusion, the LIU value obtained by the NOVA view system showed a good correlation with dEPT, but the eEPT by NOVA View showed a tendency to overestimate the titer. Further study is needed to figure out accurate cut-off LIU values for each titer and pattern to replace dEPT with eEPT for reducing the workload of routine ANA IIFA and standardization of the ANA titer.

## Figures and Tables

**Table 1:** Patient characteristics.

	Total *n* = 1518 (%)	Autoimmune Disease *n* = 697 (%)	Non-Autoimmune Disease *n* = 821 (%)	*p* Value
Age, year [1st–3rd interquartile range]	57.0 [41.0–67.0]	53.0 [39.0–64.0]	59.0 [43.0–70.0]	<0.001
Female	1161 (76.5)	589 (84.5)	572 (69.7)	<0.001
Underlying disease				
Hypertension	92 (6.1)	43 (6.2)	59 (7.2)	0.493
Diabetes mellitus	64 (4.2)	28 (4.0)	36 (4.4)	0.820
Chronic kidney disease	81 (5.3)	25 (3.6)	56 (6.8)	0.007
Malignancy	53 (3.5)	8 (1.1)	45 (5.5)	<0.001
Autoantibody Pattern				0.019
Homogeneous	632 (41.6)	265 (38.0)	367 (44.7)	
Speckled	669 (44.1)	319 (45.8)	350 (42.6)	
Nucleolar	68 (4.5)	29 (4.2)	39 (4.8)	
Centromere	129 (8.5)	73 (10.5)	56 (6.8)	
Nuclear dots	20 (1.3)	11 (1.6)	9 (1.1)	
Endpoint titer of autoantibody by dilution				<0.001
1:80	786 (51.8)	248 (35.6)	538 (65.5)	
1:160	308 (20.3)	145 (20.8)	163 (19.9)	
1:320	151 (9.9)	87 (12.5)	64 (7.8)	
≥1:640	273 (18.0)	217 (31.1)	56 (6.8)	

**Table 2:** Concordance rates between endpoint titer by serial dilution and estimated endpoint titer by the NOVA View automated ANA IIFA system.

	Total (*n* = 1518)	Speckled Pattern (*n* = 669)	Homogeneous Pattern (*n* = 632)	Centromere Pattern (*n* = 129)	Nucleolar Pattern (*n* = 68)	Nuclear Dot Pattern (*n* = 20)
Before adjustment						
Exact match, *n* (%)	336 (22.1)	213 (31.8)	58 (9.2)	79 (61.2)	10 (14.7)	10 (50.0)
±One-titer match, *n* (%)	699 (46.0)	402 (60.1)	200 (31.6)	81 (62.8)	38 (55.9)	12 (60.0)
Categorical match, *n* (%)	780 (51.4)	345 (51.6)	259 (41.0)	119 (92.3)	49 (61.2)	16 (80.0)
After downgraded adjustment of eEPT *						
Exact match, *n* (%)	774 (51.0)	290 (43.4)	353 (66.4)	87 (67.4)	29 (42.7)	9 (45.0)
±One-titer match, *n* (%)	1412 (93.0)	576 (86.1)	500 (94.0)	124 (96.1)	61 (89.7)	16 (80.0)
Categorical match, *n* (%)	1267 (83.5)	579 (86.6)	397 (74.6)	121 (93.8)	49 (72.1)	18 (90.0)

* Two-titer downgraded adjustment was evaluated in speckled, homogeneous nucleolar, and nuclear dot patterns, and three-titer downgraded adjustment was evaluated in centromere pattern.

## Data Availability

The data that support the findings of this study are available from the corresponding author upon reasonable request.

## Abbreviations

ANA, Antinuclear antibody; IIFA, indirect immunofluorescence assay; eEPT, estimated endpoint titer; dEPT, endpoint titer by dilution; LIU, light intensity unit; FITC, fluorescein-5-isothiocyanate; DAPI, 4′-6-diamidino-2-phenylindol; IQR, interquartile range.

## References

[1] Agmon-Levin N., Damoiseaux J., Kallenberg C., Sack U., Witte T., Herold M., Bossuyt X., Musset L., Cervera R., Plaza-Lopez A. (2014). International recommendations for the assessment of autoantibodies to cellular antigens referred to as anti-nuclear antibodies. Ann. Rheum. Dis..

[2] Damoiseaux J., Andrade L.E.C., Carballo O.G., Conrad K., Francescantonio P.L.C., Fritzler M.J., Garcia de la Torre I., Herold M., Klotz W., de Melo Cruvinel W. (2019). Clinical relevance of HEp-2 indirect immunofluorescent patterns: The International Consensus on ANA patterns (ICAP) perspective. Ann. Rheum. Dis..

[3] Barbara D., John L.S., Robert G.H. (2016). Manual of Molecular and Clinical Laboratory Immunology.

[4] Meroni P.L., Schur P.H. (2010). ANA screening: An old test with new recommendations. Ann. Rheum. Dis..

[5] Tozzoli R., Bonaguri C., Melegari A., Antico A., Bassetti D., Bizzaro N. (2013). Current state of diagnostic technologies in the autoimmunology laboratory. Clin. Chem. Lab. Med..

[6] Tebo A.E. (2017). Recent Approaches To Optimize Laboratory Assessment of Antinuclear Antibodies. Clin. Vaccine. Immunol..

[7] Bossuyt X., Hendrickx A., Frans J. (2005). Antinuclear antibody titer and antibodies to extractable nuclear antigens. Ann. Rheum. Dis..

[8] Pham B.N., Albarede S., Guyard A., Burg E., Maisonneuve P. (2005). Impact of external quality assessment on antinuclear antibody detection performance. Lupus.

[9] Copple S.S., Giles S.R., Jaskowski T.D., Gardiner A.E., Wilson A.M., Hill H.R. (2012). Screening for IgG antinuclear autoantibodies by HEp-2 indirect fluorescent antibody assays and the need for standardization, *Am*. J. Clin. Pathol..

[10] Tozzoli R., Antico A., Porcelli B., Bassetti D. (2012). Automation in indirect immunofluorescence testing: A new step in the evolution of the autoimmunology laboratory. Auto. Immun. Highlights.

[11] Mahler M., Meroni P.L., Bossuyt X., Fritzler M.J. (2014). Current concepts and future directions for the assessment of autoantibodies to cellular antigens referred to as anti-nuclear antibodies. J. Immunol. Res..

[12] Meroni P.L., Bizzaro N., Cavazzana I., Borghi M.O., Tincani A. (2014). Automated tests of ANA immunofluorescence as throughput autoantibody detection technology: Strengths and limitations. BMC Med..

[13] Ricchiuti V., Adams J., Hardy D.J., Katayev A., Fleming J.K. (2018). Automated Processing and Evaluation of Anti-Nuclear Antibody Indirect Immunofluorescence Testing. Front. Immunol..

[14] Choi H.W., Kwon Y.J., Park J.H., Lee S.Y., Chun S., Won E.J., Lee J.H., Choi H.J., Kim S.H., Shin M.G. (2020). Evaluation of a Fully Automated Antinuclear Antibody Indirect Immunofluorescence Assay in Routine Use. Front. Immunol..

[15] Bizzaro N., Antico A., Platzgummer S., Tonutti E., Bassetti D., Pesente F., Tozzoli R., Tampoia M., Villalta D. (2014). Automated antinuclear immunofluorescence antibody screening: A comparative study of six computer-aided diagnostic systems. Autoimmun. Rev..

[16] Zheng B., Li E., Zhu H., Lu J., Shi X., Zhang J., Li M. (2017). Automated antinuclear immunofluorescence antibody analysis is a reliable approach in routine clinical laboratories. Clin. Chem. Lab. Med..

[17] Van Hoovels L., Schouwers S., Van den Bremt S., Bogaert L., Vandeputte N., Vercammen M., Bossuyt X. (2018). Analytical performance of the single well titer function of NOVA View^®^: Good enough to omit ANA IIF titer analysis?. Clin. Chem. Lab. Med..

[18] Van den Bremt S., Schouwers S., Van Blerk M., Van Hoovels L. (2017). ANA IIF Automation: Moving towards Harmonization? Results of a Multicenter Study. J. Immunol. Res..

[19] Schouwers S., Bonnet M., Verschueren P., Westhovens R., Blockmans D., Mariën G., Bossuyt X. (2014). Value-added reporting of antinuclear antibody testing by automated indirect immunofluorescence analysis. Clin. Chem. Lab. Med..

[20] Bonroy C., Verfaillie C., Smith V., Persijn L., De Witte E., De Keyser F., Devreese K. (2013). Automated indirect immunofluorescence antinuclear antibody analysis is a standardized alternative for visual microscope interpretation. Clin. Chem. Lab. Med..

[21] Roggenbuck D., Hiemann R., Schierack P., Reinhold D., Conrad K. (2014). Digital immunofluorescence enables automated detection of antinuclear antibody endpoint titers avoiding serial dilution. Clin. Chem. Lab. Med..

[22] Yoo I.Y., Oh J.W., Cha H.S., Koh E.M., Kang E.S. (2017). Performance of an Automated Fluorescence Antinuclear Antibody Image Analyzer. Ann. Lab. Med..

[23] Carbone T., Gilio M., Padula M.C., Tramontano G., D’Angelo S., Pafundi V. (2018). A step towards standardization: A method for end-point titer determination by fluorescence index of an automated microscope. End-point titer determination by fluorescence index. J. Immunol. Methods.

